# Factors Associated with Replacement Interval and Complications of Provox Voice Prostheses in Post-Laryngectomy Patients: A Single-Center Retrospective Recurrent-Event Analysis

**DOI:** 10.3390/cancers18162545

**Published:** 2026-08-07

**Authors:** Dominik Pawlicki, Marta Gamrot-Wrzoł, Olga Karłowska-Bijak, Tomasz Stącel, Michał Napierała, Maciej Misiołek, Paweł Sowa

**Affiliations:** 1 Department of Otorhinolaryngology and Oncological Laryngology, Faculty of Medical Sciences in Zabrze, Medical University of Silesia in Katowice, 40-055 Zabrze, Poland; 2 Cardiothoracic Department and Cardiovascular Research Department, Corewell Health Butterworth Hospital, Grand Rapids, MI 49503, USA; 3 Faculty of Medical Sciences, Medical University of Silesia in Katowice, 40-055 Katowice, Poland

**Keywords:** voice prosthesis, Provox, total laryngectomy, tracheoesophageal prosthesis, voice rehabilitation, laryngeal cancer, radiotherapy, chemoradiotherapy, device survival

## Abstract

**Background/Objectives**: Indwelling tracheoesophageal prostheses have a limited lifespan and require repeated replacement. We examined the determinants of the replacement interval. **Methods**: This study conducted a retrospective single-center analysis of 147 consecutive Provox replacements in 82 laryngectomized patients (June 2024–February 2026), reported according to STROBE, using mixed-effects and gap-time recurrent-event models. **Results**: Median device survival was 141 days. Prior irradiation was associated with a shorter interval (−38.3 days; 95% CI −67.2 to −9.3; *p* = 0.010), with a gradient by treatment intensity: the median survival was 179, 136, and 84 days after primary surgery, radiotherapy alone, and chemoradiotherapy (log-rank *p* < 0.001; cluster-robust hazard ratios 2.19 and 5.13 against primary surgery). A higher cumulative replacement burden—in terms of lifetime exchanges per patient, with a median of eight and range of 1–28—was also associated with shorter intervals (β = −3.70 days per additional lifetime exchange; 95% CI −5.84 to −1.57), but it summarizes the same event process as the outcome and is reported as a phenotype marker. Intervals ending in elective exchange were longer (β = 35.47 days; 95% CI 15.80 to 55.14), but in the primary recurrent-event model, containing only covariates fixed at interval start and retaining censored intervals, elective placement showed no association with device lifetime (HR 0.574; 95% CI 0.309–1.066; *p* = 0.079); between-patient heterogeneity remained substantial (θ = 0.452; *p* = 0.002). Complications occurred in seven of 147 procedures (4.8%), which were all managed without life-threatening events. **Conclusions**: Prior oncological treatment, particularly chemoradiotherapy, is associated with materially shorter device survival and can be used to identify patients likely to require more frequent exchange. Outpatient exchange appears safe. The association between elective replacement and longer intervals reflects conditioning inherent in comparing scheduled with failure-driven exchange, not a benefit of scheduling.

## Simple Summary

After total laryngectomy for advanced laryngeal cancer, voice is most often restored with an indwelling tracheoesophageal prosthesis. These silicone devices have a limited lifespan and must be replaced repeatedly. This single-center retrospective study reviewed our experience with Provox replacements and compared planned exchanges with unscheduled, leakage-driven ones. Among 82 patients undergoing 147 replacements, those with a higher lifetime number of replacements had shorter intervals between exchanges, which identifies who replaces often rather than explaining why. Intervals ending in a planned exchange were longer than those ending in an unplanned one, but that comparison is defined in part by its own outcome, and an analysis designed to avoid this circularity found no such advantage. These data therefore do not support the view that scheduled replacement prolongs device life. What they do show is that patients treated with radiotherapy, and especially with chemoradiotherapy, before laryngectomy need their prosthesis replaced considerably sooner, and that outpatient exchange is safe.

## 1. Introduction

Laryngeal cancer continues to pose a significant global health burden. Recent worldwide estimates indicate that it ranked among the most common malignancies in 2022, ranking 18th among cancer-related causes of death and as the 20th most common cancer [1]. These GLOBOCAN 2022 [1] estimates remain the most recent comprehensive global figures available, as such estimates are compiled and updated only periodically. Since the first total laryngectomy, performed by Theodor Billroth in 1873, loss of natural voice has remained one of the most challenging consequences of this surgical treatment [2].

Surgical voice rehabilitation advanced substantially in 1972, when the Polish laryngologist Professor Erwin Mozolewski first described a semi-permanent tracheoesophageal voice prosthesis made of silicone with a one-way valve. Although initially published in Polish and long under-recognized internationally, this work predated the widely known Blom–Singer prosthesis introduced in 1980 and laid the foundation for current tracheoesophageal voice restoration techniques [3,4,5]. The subsequent development of tracheoesophageal puncture combined with a one-way valve prosthesis represented a major breakthrough, allowing many patients to regain intelligible speech when other rehabilitation methods proved insufficient [6,7].

Early indwelling devices, followed by more advanced low-resistance models such as Provox, have demonstrated reliable performance—including lower airflow resistance, more consistent phonation, and favorable long-term device survival—in comparative clinical evaluations [8]. The principal factor limiting the long-term function of silicone prostheses is microbial colonization, which leads to valve incompetence and eventual leakage. While transprosthetic leakage is the most frequent trigger for replacement, fistula-related complications—including tract widening, stenosis, inflammation, or granulation—often require more complex management strategies [9,10,11].

In our department, routine Provox-based voice rehabilitation was implemented in 2020, accompanied by standardized documentation of every replacement procedure from the beginning. Elective exchanges are typically scheduled every 3–4 months during outpatient follow-up, while unscheduled procedures are performed on the same day in cases of leakage with aspiration risk. Both elective and unscheduled replacements are performed as day-case procedures under local anesthesia, with pre-procedural blood tests (complete blood count, INR, APTT) and same-day discharge when no complications arise. General anesthesia is reserved only for the rare secondary interventions described in the Complications Section 3.2. (e.g., re-insertion after fistula closure).

Despite the maturity of this technique, the working life of an indwelling prosthesis remains short and highly variable. The reported mean device lifetimes cluster in the region of three to six months [12], but the dispersion both between and within patients is wide, and contemporary series have described lifetimes below this historical range, attributed to the intensification of oncological treatment in the organ-preservation era [13,14]. The determinants of device life have only been partly characterized. The most consistently reported are prior irradiation and gastro-esophageal reflux, both of which retained independent significance in multivariate analysis in the largest dedicated study, with irradiated patients showing a mean in situ lifetime of 163.3 against 202.9 days [15,16]. Device design also matters, with problem-solving prostheses achieving substantially longer wear in patients selected for recurrent early failure [17]. The proximate mechanism in most cases is colonization of the silicone by a mixed bacterial and fungal biofilm, predominantly Candida species, which impairs valve closure and degrades the material itself [18,19,20].

The practical consequence is that most replacements are unscheduled, performed when the patient presents with leakage. Whether this should be replaced by scheduled prophylactic exchange remains unresolved, and the published evidence points in opposite directions. A Polish center reported a fall in tracheoesophageal fistula-related complications from 47% to 6% after moving to three-monthly replacement [21]. Conversely, modeling work at the Netherlands Cancer Institute concluded that prophylactic replacement is not feasible, because the coefficient of variation in device lifetime is too high for any fixed schedule to intercept failures efficiently; in that analysis, a median of only about 25% of leakages could be prevented, and a schedule capturing most failures would require exchange approximately every 38 days [22]. No randomized comparison has resolved the question, and real-world data describing what actually determines the interval between successive replacements in routine practice remain scarce.

This gap motivates the present study. Specifically, it is not known whether the interval between replacements varies systematically with a patient’s accumulated replacement history, whether procedures performed electively differ from those performed without prior scheduling once that history is accounted for, and how far any such difference reflects a genuine effect rather than the structure of the comparison itself.

The objective of this study was to examine our institutional experience with Provox voice prosthesis replacement. The six-year procedure log (May 2020 to March 2026) supported only the descriptive estimation of the replacement interval; all hypothesis testing used the structured-documentation dataset spanning June 2024 to February 2026. We evaluated (1) factors influencing the time interval between replacements—including the cumulative replacement burden and elective versus unscheduled status—and (2) indications for replacement, modifications in prosthesis size, and the occurrence of intra- and post-procedural complications.

## 2. Materials and Methods

This retrospective single-center study encompassed all Provox voice prosthesis replacements performed between May 2020 and March 2026 at the Department of Otorhinolaryngology and Oncological Laryngology, Specialist Hospital, Zabrze, Poland. Data were derived from hospital electronic medical records and from prospectively completed standardized Provox replacement forms. Only indwelling Provox models were used, Provox Vega (PV) and Provox Vega XtraSeal (PVXS) (Atos Medical, Malmö, Sweden), with shaft lengths ranging from 4.0 to 12.5 mm. Provox Vega ActiValve was not used in any procedure during the study period. Device selection was determined by national reimbursement policy: Atos Medical prostheses are the only indwelling voice prostheses funded by the Polish National Health Fund, and no alternative manufacturer was available to these patients. The collected parameters included patient demographics (age, sex), prosthesis type and size before and after exchange, indication for replacement (elective vs. unscheduled: transprosthetic leakage, periprosthetic leakage, aphonia, obstruction, fistula issues, other), complications, and clinical notes. All procedures were performed on an ambulatory basis by department staff, including residents and consultants. Unscheduled replacements were performed on the day of presentation when clinically indicated.

### 2.1. Study Population and Data Structure

**Figure 1:**
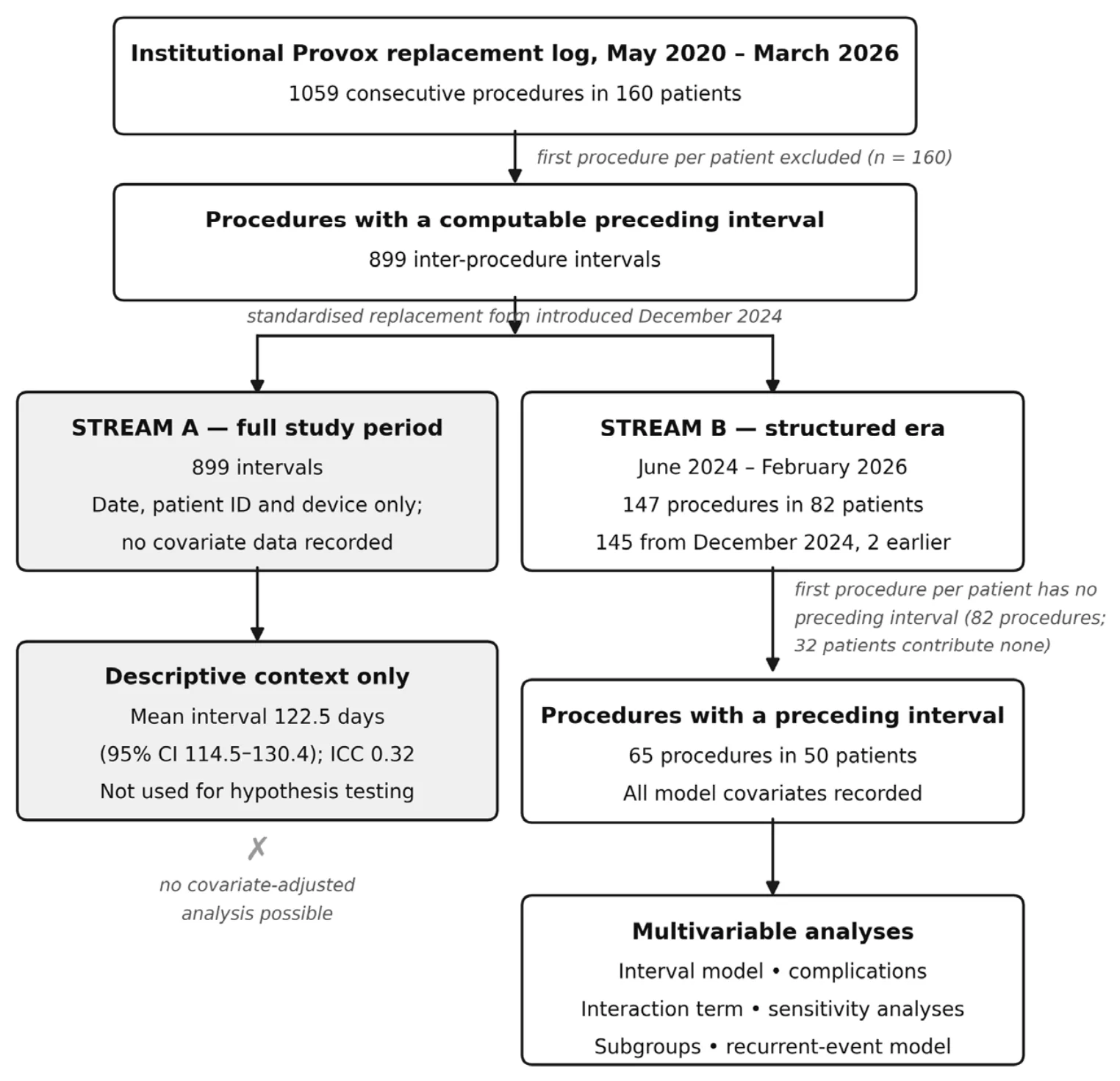
Derivation of the two analytical datasets. The institutional procedure log supported only the descriptive estimation of the replacement interval; all hypothesis testing was performed on the structured-documentation dataset.

Two datasets were used. The institutional procedure log covers May 2020 to March 2026 and comprises 1059 consecutive procedures in 160 patients, yielding 899 inter-procedure intervals; it records only the patient identifier, procedure date, and device, and therefore supports the descriptive estimation of the replacement interval but no adjusted analysis. In December 2024, a standardized replacement form was introduced, capturing the indication, prosthesis type and size before and after exchange, procedural status, and complications prospectively at the point of care. All hypothesis testing was performed on the resulting dataset of 147 consecutive procedures in 82 patients performed between June 2024 and February 2026. The form came into systematic use in December 2024, from which date 145 of the 147 procedures are derived; the two earlier procedures were documented on the same form and are retained. The dataset is a complete consecutive census rather than a sample (Figure 1). The census date for right-censoring is the date of the last procedure recorded in the structured dataset, 16 February 2026; devices in place on that date are treated as right-censored. The completed STROBE checklist is provided in the Supplementary Materials.

Two counts of replacement activity are distinguished throughout and are not interchangeable. The cumulative replacement burden is the total number of exchanges documented for a patient in the institutional log over the whole period from May 2020, including those performed before the analytical window; it takes the same value for every procedure which that patient contributes (median, 8; IQR, 5–13; mean, 8.9; SD, 5.3; range, 1–28). Procedures within the study window are the number of exchanges that the same patient contributed between June 2024 and February 2026 (median, 2; IQR, 1–2; mean, 1.8; SD, 0.8; range, 1–4). The two coincide in only 7 of the 82 patients. The cumulative replacement burden is the variable entered into every model in this paper; the within-window count is descriptive and is never modeled. The cumulative burden is a patient-level characteristic rather than a running count preceding each exchange, so associations involving it are between patients rather than within them.

No patient or procedure entered the analytical dataset on the basis of any characteristic; the boundary between datasets is the date on which structured documentation began. Two limitations follow. A window opening in December 2024 preferentially captures patients already established on prosthetic rehabilitation, so the observed complication rate is a lower bound; the extent of this left truncation is quantified in Section 3.6. For patients whose first insertion preceded December 2024, the replacement burden was reconstructed from the log and may be understated. Procedures were performed by 14 physicians, three of whom accounted for 68% of the total.

See Table 1 for a summary of the baseline characteristics of the study population.

Prior oncological treatment, smoking history, and proton-pump inhibitor therapy were available for all 82 patients: 25 (30.5%) had received radiotherapy alone before laryngectomy, 14 (17.1%) had received chemoradiotherapy, and 43 (52.4%) had received primary surgery without irradiation, giving 39 (47.6%) with any prior irradiation; 47 (57.3%) had a smoking history, and 18 (22.0%) were receiving proton-pump inhibitor therapy for reflux. These are patient-level characteristics, invariant across a patient’s procedures, and were entered into the models on that basis.

### 2.2. Definitions of Replacement Indications

Indications were assigned at the point of care by the operating physician according to the following criteria, which were adapted from the standardized algorithm for periprosthetic leakage proposed by Parrilla et al. [23].

Central (transprosthetic) leakage: The passage of fluid through the valve lumen, observed directly during a swallow test with tinted water and the prosthesis in situ, with a dry periprosthetic margin.

Peripheral (periprosthetic) leakage: The passage of fluid around the external circumference of the prosthesis shaft, between the device and the fistula wall, with a competent valve during the same swallow test.

Prosthesis dysfunction without phonation: The inability to produce voice despite correct digital occlusion of the tracheostoma, with a patent and correctly seated device upon inspection.

Elective replacement: The exchange performed at a pre-arranged outpatient appointment for a functioning device, irrespective of device age. Unscheduled replacement: The exchange performed on the day of unscheduled presentation for any of the above indications.

Where central and peripheral leakage coexisted, the indication judged primarily by the operating physician was recorded; the recording scheme did not permit dual coding, which may have reduced the apparent frequency of mixed presentations. No formal assessment of inter-rater reliability was undertaken, and agreement between the 14 contributing physicians cannot therefore be quantified.

### 2.3. Statistical Analysis

Analyses used mixed-effects models with a random intercept for patients, accounting for repeated procedures within individuals. Linear models were fitted to the interval between replacements and logistic models to binary outcomes; the intraclass correlation quantified between-patient variance. Estimates are reported with 95% confidence intervals, with significance at *p* < 0.05. Analyses were performed in Stata 19.5 and Python 3.12.

Because the interval model rests on 65 procedures in 50 patients, simultaneous adjustment for all candidate confounders would result in overfitting. A pre-specified parsimonious model was retained (replacement burden, procedural status, age, and sex), with each further available confounder entered singly; the coefficient’s stability is reported in Table 2. Prior oncological treatment, smoking history, and proton-pump inhibitor therapy are patient-level characteristics and were entered into a separate model reported in Section 3.8; because replacement burden is also patient-level and is correlated with prior irradiation, the two were not adjusted for one another in the primary specification.

Sensitivity analyses comprised the exclusion of frequent replacers (pre-specified as four or more replacements within any 12-month period, or two successive replacements less than three months apart, matching the eligibility criteria for registered trials of problem-solving prostheses), leave-one-patient-out estimation, winsorization at the 5th and 95th percentiles, adjustment for the duration of prosthetic rehabilitation, and adjustment for operator volume. Subgroup analyses were pre-specified for age, sex, and prosthesis type using interaction terms, with strata of fewer than ten procedures not analyzed.

Because procedural status is determined at the end of an interval rather than at its start, an interval ending in an elective exchange is, by construction, one in which the device survived to a scheduled appointment. The analysis was therefore repeated as a recurrent-event problem in gap time, with every covariate measured at the beginning of each interval. In that formulation, all 147 procedures contribute, terminating either in a subsequent replacement (65 events) or in right-censoring at the census date (81 censored intervals), which the linear model discards. A Weibull proportional-hazards model with a gamma-distributed shared frailty was fitted by maximum marginal likelihood, the likelihood-ratio test for the frailty variance evaluated against the 50:50 mixture null; device survival was summarized by the Kaplan–Meier estimator. Because the Kaplan–Meier estimator and the log-rank test treat devices as independent, associations with prior oncological treatment were additionally estimated on the same intervals in a Cox proportional-hazards model with a cluster-robust sandwich variance grouped by patient, and in the shared-frailty specification described above; the two treatment terms were tested jointly with the Wald test. An interaction between procedural status and replacement burden was tested and is reported as exploratory.

The interval distribution was close to symmetric (skewness −0.09), and residuals showed no evidence against normality (Shapiro–Wilk W = 0.981, *p* = 0.421). Logarithmic transformation degraded the fit (W = 0.848, *p* < 0.001) and was not used.

## 3. Results

### 3.1. Time Interval Between Replacement Procedures

A mixed-effects linear regression model with a random intercept for each patient was used to evaluate factors associated with the time interval between procedures. Data on procedure dates were available for the entire historical patient cohort (*n* = 1059 procedures; 899 intervals from May 2020 to March 2026); in the null model, the average interval between prosthesis replacements was 122.5 days (95% CI: 114.5–130.4), with substantial between-patient variability (variance of the random intercept = 1481.7) and an intraclass correlation coefficient (ICC) of 0.32 (95% CI: 0.24–0.42).

Within the specific study sample analyzed in detail here (147 procedures in 82 patients, June 2024–February 2026), the null model estimated a mean interval of 117.3 days (95% CI: 103.5–131.0), again with substantial between-patient variability (variance of the random intercept = 1462.8) and a higher ICC of 0.55 (95% CI: 0.25–0.82), indicating that approximately 55% of the total variability in the replacement interval is attributable to between-patient differences.

In the multivariable model (*n* = 65 procedures in 50 patients), only two factors were significantly associated with the interval between procedures (Table 2). The reduction from 147 procedures to 65 is structural rather than a consequence of missing data: an interval can be computed only for a procedure preceded by another within the observation window, and 82 procedures were each patient’s first within that window. Thirty-two patients contributed a single procedure and therefore had no interval. No covariate values were missing for any procedure. A higher cumulative replacement burden per patient was associated with a shorter interval (β = −3.70 days per additional lifetime exchange; 95% CI −5.84 to −1.57; *p* = 0.001). Because this variable is a patient-level characteristic, this association is between patients and does not by itself demonstrate progressive shortening within individuals; the within-patient analysis is reported in Section 3.4. In contrast, planned procedures were associated with longer intervals (β = 35.47 days; 95% CI: 15.80 to 55.14; *p* < 0.001).

The between-patient variability remained substantial in the adjusted model (random intercept variance = 796.13), and the likelihood-ratio test confirmed that the mixed-effects model provided a better fit than a standard linear model (*p* = 0.007).

Among the tested but non-significant covariates, gender was not a significant predictor of the interval between events in the mixed-effects model (β = 5.73, *p* = 0.567), although a simple t-test indicated a significant difference (mean difference = 17.05 days, *p* = 0.0025). The mixed-effects estimate is considered more appropriate for these repeated-measures data.

### 3.2. Complications

Complications occurred in seven of the 147 voice prosthesis replacement procedures (4.8%); these represent intra- or post-procedural adverse events related to the exchange itself, distinct from leakage and aphonia, which were classified as indications for replacement rather than complications. The complications observed included granulation tissue formation (*n* = 2), prosthesis displacement within the fistula (*n* = 1), prosthesis displacement with subsequent tracheoesophageal fistula closure (*n* = 2), and swallowing of the prosthesis (*n* = 2).

Granulation tissue formation (*n* = 2) required surgical removal under local anesthesia followed by systemic antibiotic therapy (amoxicillin–clavulanate for 7 days). Prosthesis displacement was noted in three cases. In one case, the prosthesis was successfully removed and replaced with a new one during the same outpatient procedure. In the remaining two cases, displacement led to closure of the tracheoesophageal fistula, necessitating referral for secondary voice prosthesis insertion under general anesthesia. Swallowing of the prosthesis occurred in two patients. The first felt the device leave the fistula during a bout of coughing while eating at midday and presented the following morning; the second did not observe the device leave and presented after noticing leakage, also the following morning. In both, posteroanterior and lateral radiography of the chest and abdomen was obtained on the morning of presentation, approximately 18 to 24 h after the device was last known to be in place, and showed no prosthesis in the airway or the gastrointestinal tract. Provox prostheses carry a radiopaque valve seat and are therefore visible radiographically. Because imaging was obtained on the day after the event rather than at the time of it, the negative films exclude a device retained in the airway at the moment of examination but cannot establish its location during the preceding hours; complete gastrointestinal transit within that interval is plausible. Neither prosthesis was recovered, so its passage is inferred rather than demonstrated. Both patients remained free of respiratory or abdominal symptoms at every subsequent outpatient visit. No life-threatening complications were recorded. The low complication rate (4.8%) is consistent with a favorable safety profile of outpatient voice prosthesis exchange in a clinical ENT department.

All seven events occurred in male patients, in a cohort that is 74% male; this co-occurrence was not tested statistically and does not identify a risk factor. With seven events in total, no inferential model was fitted to complications, with events per variable being below one, and they are presented descriptively only. Five followed unscheduled procedures (five of 73; 6.8%), and two followed elective procedures (two of 74; 2.7%). A change in device relative to the one removed occurred in 31 of the 65 procedures for which the removed device was identifiable (47.7%), and a change in type between Vega and Vega XtraSeal occurred in 13 (20.0%). No inferential model of size change is reported: with 65 informative procedures, a multivariable logistic model would carry fewer than five events per candidate covariate, and we judged it uninformative. Size modification is therefore described rather than modeled, and the corresponding objective is framed descriptively.

### 3.3. Interaction Between Procedural Status and Replacement History

The interaction between procedural status and cumulative replacement burden was not statistically significant (β = −2.15 days; 95% CI −7.16 to 2.86; *p* = 0.401), providing no evidence that the association between elective replacement and a longer subsequent interval differs according to a patient’s overall replacement burden. In this model, the main effects were β = −3.27 days per additional lifetime exchange (95% CI −5.76 to −0.77; *p* = 0.010) for the replacement burden and +56.86 days (95% CI 5.04 to 108.69; *p* = 0.032) for the elective status. Because the replacement burden was not centered, the latter estimate refers to a patient with a replacement burden of zero, a value that does not occur in the data (observed minimum of one; the cumulative replacement burden is defined in Section 2.1), and should not be read as the effect of the elective status at a typical burden. Given that this analysis rests on 65 procedures in 50 patients, the non-significant interaction constitutes weak evidence of homogeneity rather than evidence of it, and the term is reported as exploratory.

### 3.4. Sensitivity Analyses

The association between replacement burden and interval was stable across sensitivity specifications (Table 3). Leave-one-patient-out estimates ranged from −4.55 to −3.32 days, and all 50 were negative; winsorization gave −3.39 days (95% CI −5.43 to −1.34; *p* = 0.001) and adjustment for duration of prosthetic rehabilitation did not attenuate it (−5.10 days; 95% CI −8.86 to −1.33; *p* = 0.008), excluding the possibility that it merely reflects longer follow-up permitting more procedures; the point estimate moved away from the null, which, together with the null coefficient for duration itself (Section 3.6), indicates collinearity between duration of rehabilitation and replacement burden rather than genuine strengthening; and adjustment for operator volume left it unchanged (−3.95 days; *p* < 0.001), operator volume itself being unassociated with the interval (*p* = 0.235).

Two analyses qualify these findings. Sixteen patients met the frequent-replacer definition through its interval criterion: two successive replacements less than three months apart; excluding them left 41 procedures in 34 patients, in which neither association remained significant (burden −1.97 days, 95% CI −4.67 to 0.72, *p* = 0.151; elective status +13.38 days, 95% CI −5.26 to 32.02, *p* = 0.160). One further patient met the frequency criterion of four or more replacements within a 12-month period without meeting the interval criterion; additionally, excluding that patient leaves 38 procedures in 33 patients and does not alter the conclusion, with neither association approaching significance. This exclusion, however, removes variance from the exposure itself: its standard deviation fell by 23% and the minimum detectable effect rose to 3.85 days, exceeding the full-sample estimate of 3.70 days; therefore, a null result was expected irrespective of whether the association was real.

A further analysis addresses the direction of conditioning. In the primary model, the interval is regressed on the status of the procedure that ended it; in contrast, modeling the status of the procedure that placed the device is prospective with respect to the interval. In that specification, elective placement was associated with an interval of 15.30 days longer, not reaching significance (95% CI −6.85 to 37.45; *p* = 0.176), while the replacement burden was similar in magnitude (−4.46 days; *p* < 0.001).

Second, because replacement burden is a patient-level constant, no within-patient decomposition can be estimated. A genuine within-patient test is possible only among the 13 patients contributing two or more observed intervals: each successive replacement was associated with an interval 4.61 days shorter (95% CI −25.92 to 16.70; *p* = 0.672) across 28 intervals, consistent in direction but too imprecise to confirm or exclude progression.

### 3.5. Subgroup Analyses

**Figure 2:**
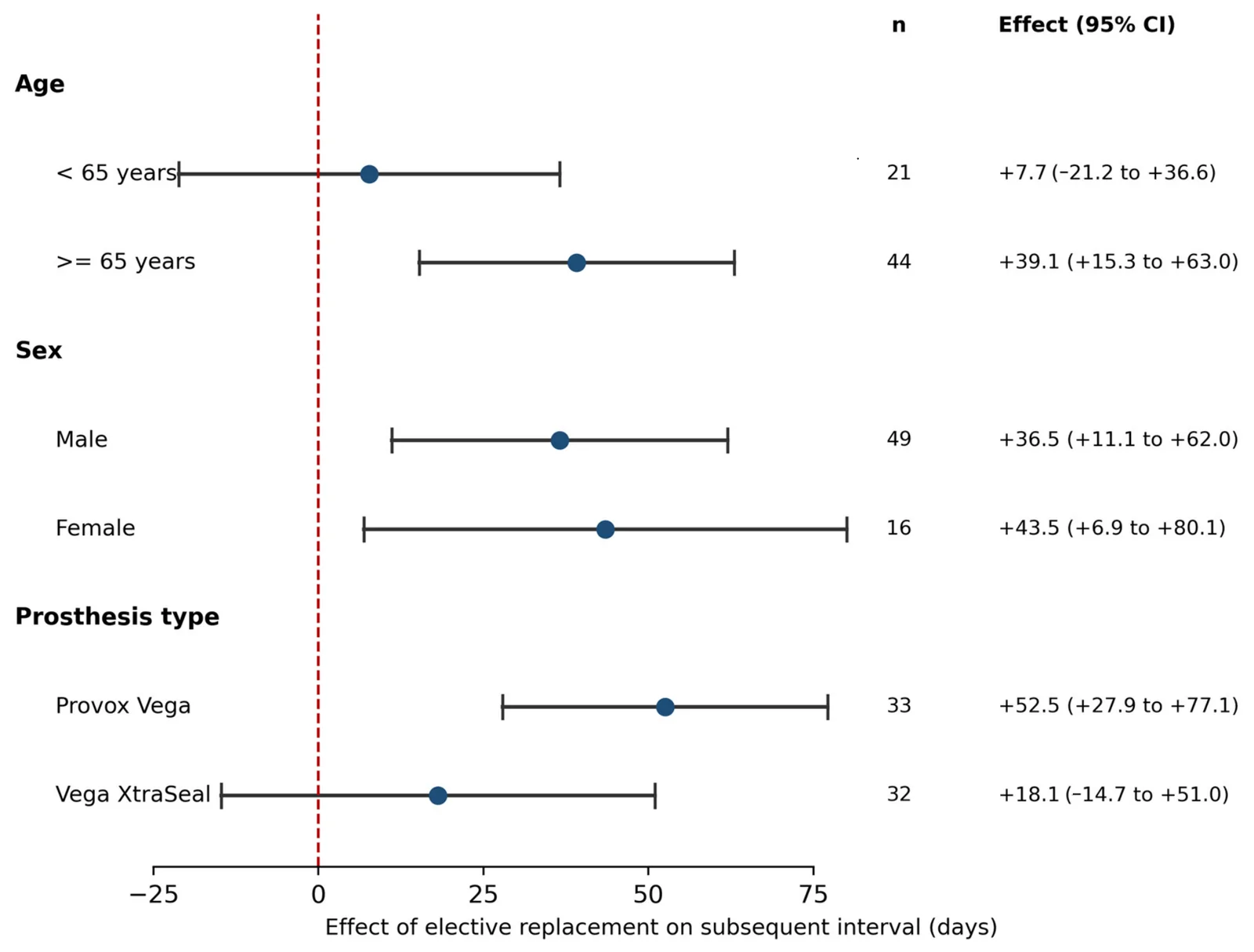
Effect of elective replacement on the subsequent interval, by subgroup (exploratory). Points are stratum-specific estimates with 95% confidence intervals; no interaction term reached statistical significance.

Subgroup analyses are presented in Figure 2. No significant interaction was observed between procedural status and age dichotomized at 65 years (*p* = 0.728), sex (*p* = 0.688), or prosthesis type (*p* = 0.106). The stratum-specific estimates nonetheless varied: the elective effect was +7.7 days (95% CI −21.2 to 36.6) in patients under 65 and +39.1 days (95% CI 15.3 to 63.0) in those aged 65 or over, and +52.5 days (95% CI 27.9 to 77.1) for Provox Vega versus +18.1 days (95% CI −14.7 to 51.0) for Vega XtraSeal. Because the corresponding interaction terms were not significant, these differences should not be interpreted as evidence that the effect of elective replacement differs between strata; they are reported to illustrate the imprecision of stratum-specific estimation at this sample size. Prior oncological treatment is analyzed separately in Section 3.8. No adjustment was made for multiple comparisons, and all the subgroup findings are exploratory.

### 3.6. Quantification of Left Truncation

Because the structured dataset began in December 2024, most patients had already been receiving prosthetic voice rehabilitation for some time when they entered it. At their first procedure within the study window, patients had been using a voice prosthesis for a median of 22 months (IQR, 9–38; range, 0–66). Only 11 of 82 patients (13.4%) entered the dataset at or near their first insertion, so 86.6% were prevalent rather than incident users. This quantifies the left truncation described in Section 2.1 and confirms that the cohort is enriched for patients established on prosthetic rehabilitation.

To assess whether this enrichment introduced systematic bias, the duration of prosthetic rehabilitation at entry was added to the multivariable interval model. It showed no association with the replacement interval (β = +0.04 days per additional month; 95% CI −1.10 to 1.18; *p* = 0.949). The coefficient for elective status was unchanged, whereas the coefficient for replacement burden changed from −3.70 to −5.10 days (Section 3.4); because the duration of rehabilitation and replacement burden are strongly collinear, this shift reflects the redistribution of shared variance rather than confounding by duration. Patients entering the dataset after long prior use therefore did not differ systematically in replacement interval from those entering earlier in their prosthetic history. This does not exclude selection with respect to unmeasured characteristics, and, in particular, cannot address patients who had already abandoned a prosthetic voice or undergone fistula closure before December 2024, who are absent by construction. It does, however, argue against differential selection on the outcome analyzed here.

### 3.7. Recurrent-Event Analysis

**Figure 3:**
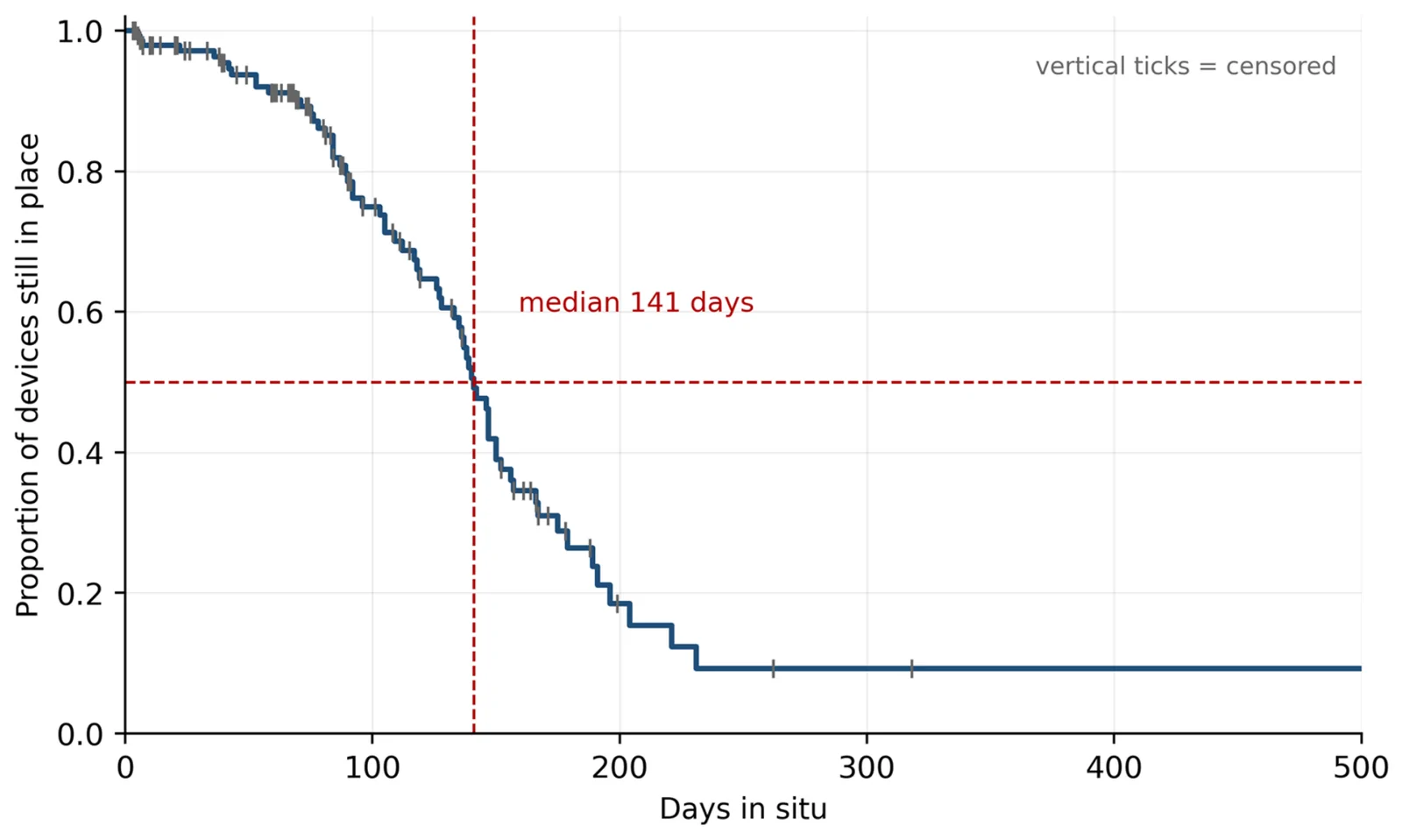
Kaplan–Meier estimate of voice prosthesis survival across the 146 intervals of positive length contributed by 147 devices, with censored intervals retained. Vertical ticks mark censored observations.

Reframing the data as a recurrent-event problem in gap time, with covariates measured at the start of each interval, uses all 147 procedures: 146 intervals of positive length, comprising 65 completed intervals and 81 that were right-censored at the census date. One patient underwent his final procedure on the census date itself, generating an interval of zero length that was excluded; 146 intervals of positive length therefore remain. The Kaplan–Meier estimate of device survival gives a median in situ lifetime of 141 days, with 78.5% of devices surviving to 90 days (95% CI 69.3–85.3), 64.7% to 120 days (95% CI 53.8–73.6), 26.4% to 180 days (95% CI 16.2–37.7), and 9.2% to 270 days (95% CI 2.7–20.7) (Figure 3). These Greenwood confidence intervals treat devices as independent and do not account for the clustering of up to four devices within a patient, so they are likely to be somewhat narrow; the Kaplan–Meier estimate is presented as descriptive. The median in situ lifetime of 141 days exceeds the mean interval of 117.3 days estimated in Section 3.1 because censored intervals, which are by construction longer than those already completed, are retained here and discarded there.

The primary specification is a Weibull proportional-hazards model with gamma shared frailty containing only covariates fixed at the start of each interval: the elective status of the procedure that placed the device, age, and sex. Cumulative replacement burden is deliberately excluded from it because the burden summarizes each patient’s entire observed event process and therefore cannot be measured at the interval start; entering it would reintroduce, in a different form, the dependence of exposure on an outcome that this analysis exists to avoid. In the primary model, elective status of the placing procedure was not associated with the subsequent interval (HR 0.574; 95% CI 0.309–1.066; *p* = 0.079), nor were age (HR 0.986; 95% CI 0.950–1.023; *p* = 0.453) or female sex (HR 1.216; 95% CI 0.595–2.487; *p* = 0.591). The estimated Weibull shape parameter was 2.354, indicating a hazard of failure that rises with time in situ, consistent with a progressive process such as biofilm accumulation. The frailty variance was substantial (θ = 0.452; likelihood-ratio test against the 50:50 mixture null, *p* = 0.002), so device lifetime clusters strongly within patients even after these covariates are accounted for. A secondary, explicitly descriptive model adds cumulative replacement burden. In it, burden is strongly associated with a shorter interval (HR 1.150 per additional lifetime exchange; 95% CI 1.096–1.207; *p* < 0.001), elective status remains unassociated (HR 0.885; 95% CI 0.499–1.572; *p* = 0.678), and the frailty variance collapses to θ = 0.031 (*p* = 0.438), a 93% reduction relative to the primary model. We do not interpret that collapse as evidence that burden explains between-patient heterogeneity: because burden is a function of the same event process that generates the outcome, the collapse is at least as readily explained as a mechanical consequence of conditioning on that process. The secondary model is reported as a description of which patients replace often, not as an estimate of an effect of regular replacement.

This analysis supersedes the linear model with respect to procedural status. When status is measured at the start of the interval rather than at its end, and when censored intervals are retained rather than discarded, no association between elective replacement and subsequent device lifetime remains. We conclude that the 35.47-day association reported in Table 2 is attributable to the structure of the comparison and not to any effect of scheduling. Cumulative replacement burden, by contrast, remains strongly associated with the interval under this reframing, but that association is descriptive: it identifies patients who replace often rather than estimating the consequence of replacing often.

**Figure 4:**
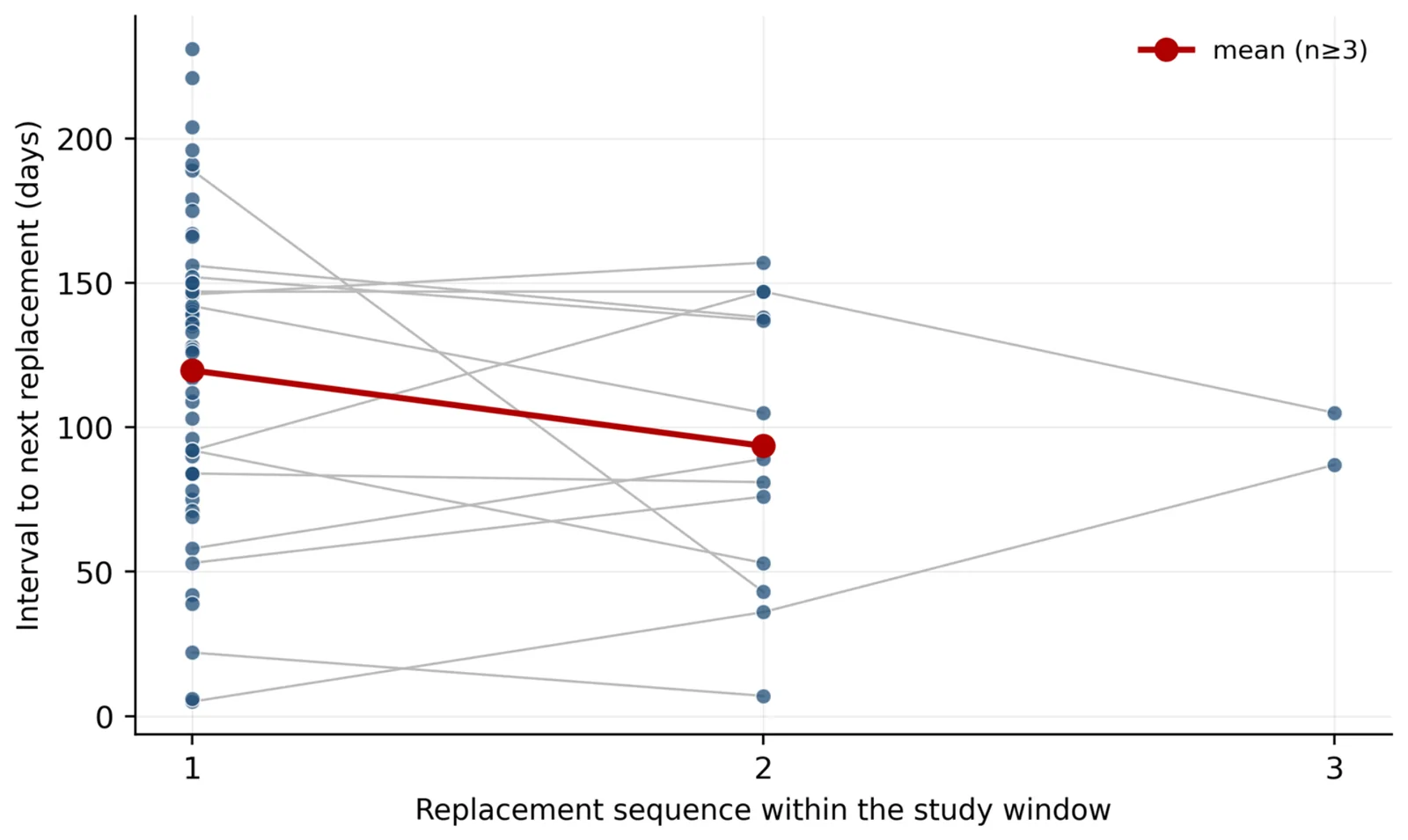
Replacement interval by within-patient sequence. Gray lines join successive intervals within a patient; the red line shows the mean at each sequence position where at least three patients contribute.

**Figure 5:**
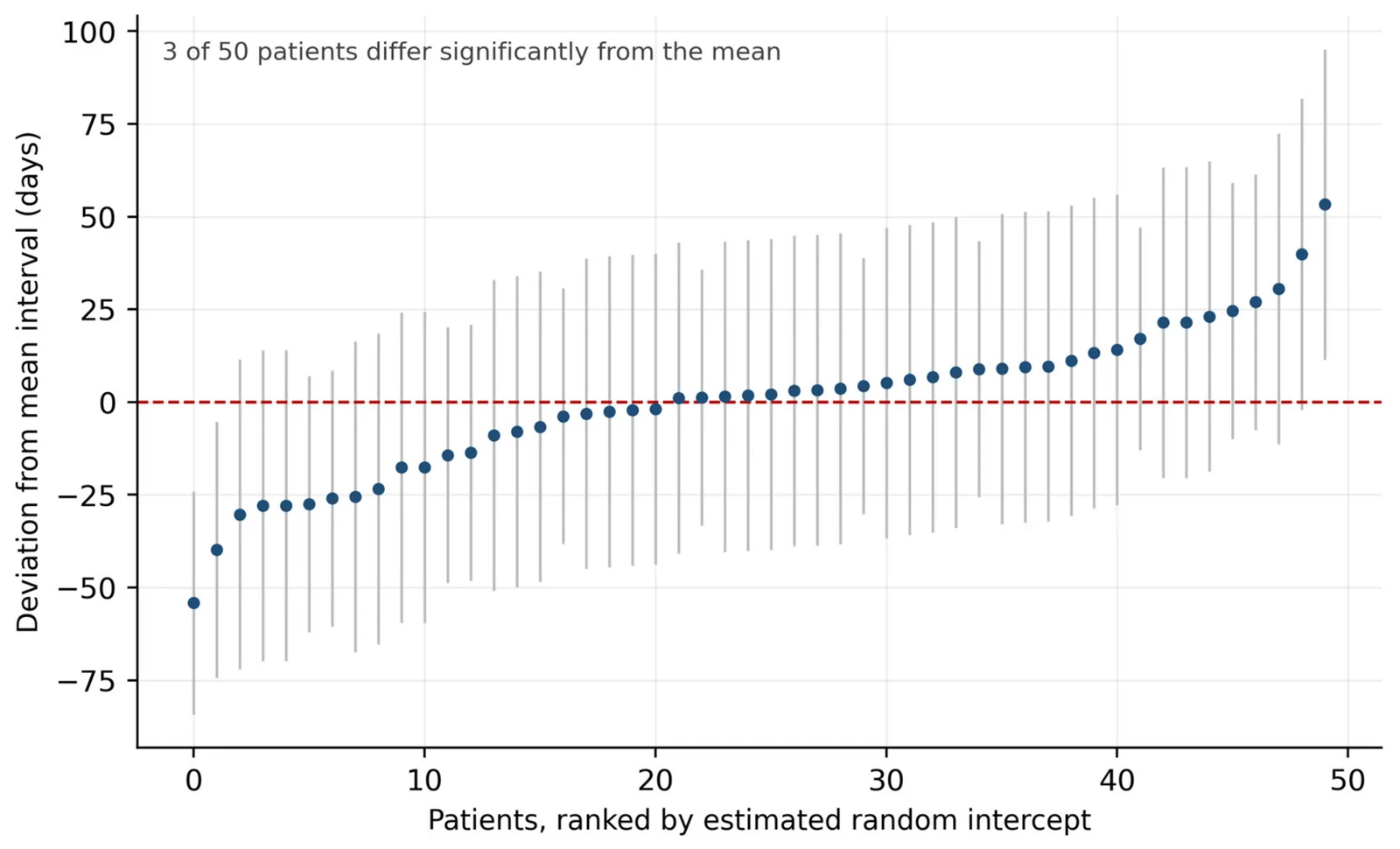
Estimated patient-level random intercepts with 95% intervals, ranked. Deviations are expressed in days relative to the cohort mean interval.

Figure 4 displays intervals against the within-patient replacement sequence, and Figure 5 shows the estimated patient-level random intercepts. The latter shows a standard deviation of 29.4 days, with only three of 50 patients differing significantly from the cohort mean, indicating that the between-patient variation detectable at this sample size is concentrated in a small minority.

### 3.8. Prior Oncological Treatment, Reflux, and Smoking

Prior oncological treatment, proton-pump inhibitor therapy, and smoking history were recorded for each patient and analyzed at that level. Among 82 patients, 39 (47.6%) had received irradiation before laryngectomy, 25 as radiotherapy alone and 14 as chemoradiotherapy.

Prior irradiation was associated with a substantially shorter replacement interval. The unadjusted mean interval was 104.7 days in irradiated patients against 137.3 days in those treated by primary surgery alone (difference −32.6 days; *p* = 0.015). Prior irradiation, proton-pump inhibitor therapy, and smoking history were entered together with age and sex in a single mixed-effects model, in which prior irradiation was associated with an interval −38.3 days shorter (95% CI −67.2 to −9.3; *p* = 0.010); the corresponding patient-level model of the mean interval gave −34.5 days (95% CI −66.2 to −2.9; *p* = 0.032). Entering the two irradiation categories separately in the same specification showed a gradient: chemoradiotherapy was associated with an interval 56.2 days shorter than primary surgery without irradiation (95% CI −88.3 to −24.0; *p* = 0.001), whereas radiotherapy alone was associated with a smaller difference that did not reach conventional significance (−24.8 days; 95% CI −55.7 to 6.1; *p* = 0.116). These interval-based estimates rest on the 50 patients contributing at least one completed interval, and that subset is not balanced with respect to exposure: 37 of 39 irradiated patients contributed an interval, compared to 13 of 43 treated by primary surgery alone, because a patient who replaces less often is less likely to accumulate a second procedure within the observation window. The comparison group is therefore restricted to the faster-replacing non-irradiated patients, which will attenuate the estimated difference. The recurrent-event analysis that follows retains all 82 patients and all 146 intervals and is not subject to this selection.

**Figure 6:**
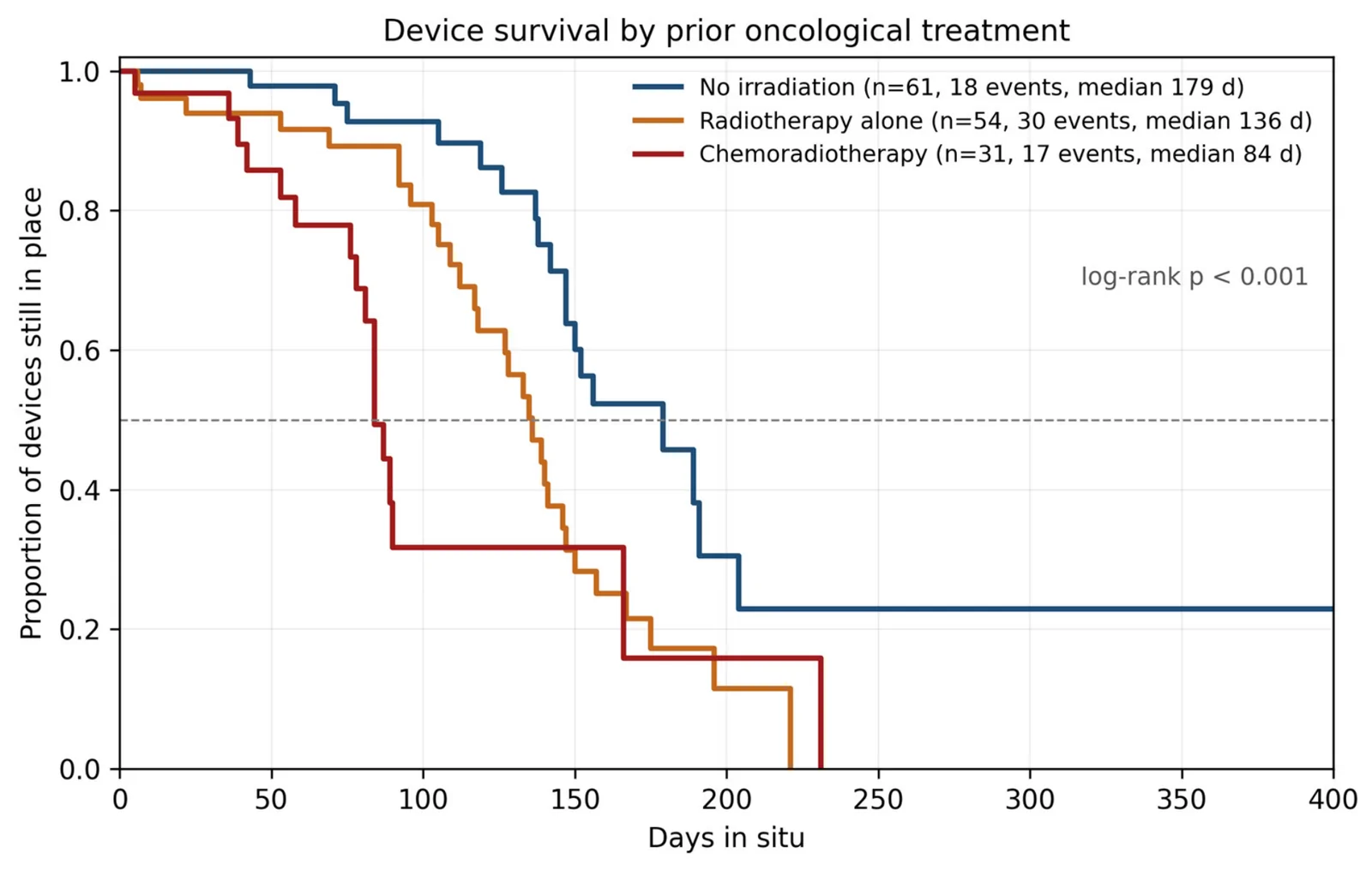
Kaplan–Meier estimate of voice prosthesis survival by prior oncological treatment. Devices in patients treated by primary surgery without irradiation are compared with those in patients who received radiotherapy alone and those who received chemoradiotherapy. Censored intervals are retained. The median in situ lifetime was 179, 136, and 84 days, respectively. Here, *n* denotes intervals of positive length, not devices or patients: 61, 54, and 31 intervals (146 in total) arising from 61, 55, and 31 devices in 43, 25, and 14 patients. The single zero-length interval excluded from the analysis falls in the radiotherapy-alone group, which is why this group contributes 30 events from 54 intervals. As in Figure 3, the Kaplan–Meier estimator and the log-rank test treat devices as independent and do not account for clustering within patients, so both are presented as descriptive; the gradient is confirmed by the cluster-robust and shared-frailty models reported in this section.

The same gradient is evident in device survival. The median in situ lifetime was 179 days without irradiation, 136 days after radiotherapy alone and 84 days after chemoradiotherapy (log-rank *p* < 0.001; Figure 6). Irradiated patients also carried a higher cumulative replacement burden, as defined in Section 2.1 (mean of 10.4 against 7.5 lifetime exchanges; *p* = 0.013), so replacement burden lies on the causal path between irradiation and interval length rather than confounding it and was not adjusted for in these models; doing so attenuated the irradiation coefficient to −16.4 days (*p* = 0.204), as expected when a mediator is conditioned upon.

Both the Kaplan–Meier estimate and the log-rank test treat devices as independent, so the gradient was re-estimated in specifications that retain the clustering of devices within patients. In a Cox proportional-hazards model of the 146 intervals with a cluster-robust variance grouped by patient, radiotherapy alone carried a hazard of replacement 2.19 times that of primary surgery without irradiation (95% CI 1.16 to 4.13; *p* = 0.015) and chemoradiotherapy carried a hazard 5.13 times as high (95% CI 2.31 to 11.39; *p* < 0.001), with no association for age (HR 0.974; 95% CI 0.944 to 1.005) or female sex (HR 1.02; 95% CI 0.51 to 2.05); the two treatment terms tested jointly gave a Wald statistic of 18.5 on two degrees of freedom (*p* < 0.001). Adding smoking history and proton-pump inhibitor therapy left the gradient intact (radiotherapy alone HR 2.27; chemoradiotherapy HR 6.19). The Weibull model with gamma shared frailty, as specified in Section 3.7, reproduced the same ordering (radiotherapy alone HR 2.97; 95% CI 1.43 to 6.17; *p* = 0.004; chemoradiotherapy HR 6.21; 95% CI 2.68 to 14.36; *p* < 0.001), and, in it, the frailty variance fell from θ = 0.452 to θ = 0.219, so prior oncological treatment accounts for approximately half of the between-patient heterogeneity in device lifetime and leaves the remainder unexplained (likelihood-ratio test against the 50:50 mixture null, *p* < 0.001). That reduction differs in kind from the collapse produced by the cumulative replacement burden in Section 3.7: prior treatment is fixed before the first prosthesis is fitted and is not a function of the event process, so the variance it removes is the variance it explains.

Neither proton-pump inhibitor therapy nor smoking history showed the association reported elsewhere. Both carried positive coefficients in that model (+42.8 days, 95% CI 7.3 to 78.3, *p* = 0.018 for proton-pump inhibitor therapy; +33.8 days, 95% CI 4.9 to 62.6, *p* = 0.022 for ever-smoking), which we do not interpret as protective. For proton-pump inhibitor therapy, the exposure is a prescription rather than endoscopically confirmed reflux, and treated reflux is the condition under active management rather than the untreated state that Boscolo-Rizzo et al. associated with a shortened lifetime [15]; Danic Hadzibegovic et al. likewise found that proton-pump inhibitor therapy alters the occurrence of prosthesis complications [24]; the coefficient is at least as consistent with effective treatment as with the absence of an effect. Moreover, both coefficients are confined to the irradiated stratum: among patients treated by primary surgery alone, the intervals were 137.8 days in ever-smokers versus 136.2 in never-smokers and 126.0 days with proton-pump inhibitor therapy versus 137.9 without, whereas the entire apparent positive association arises among irradiated patients. An association present in one stratum only, in the direction opposite to that predicted, is more readily explained by residual differences in the composition of that stratum than by an effect of either exposure. The confidence intervals convey the precision available: with 18 patients receiving proton-pump inhibitor therapy and 47 with a smoking history, both intervals remain wide enough to include differences in the magnitude reported for reflux in [15]. We report these estimates for completeness and draw no inference from them.

## 4. Discussion

This retrospective study of 82 laryngectomized patients undergoing 147 voice prosthesis replacements identifies two associations of potential clinical relevance and shows that only one of them survives an analysis in which exposure is not defined by the outcome.

In the secondary descriptive model of Section 3.7, the cumulative replacement burden corresponds to a 15% higher hazard of replacement per additional lifetime exchange. Against a median device survival of 141 days, this is large enough to be recognizable in clinical practice, but it describes which patients undergo frequent replacement rather than a consequence of replacing often. No minimal clinically important difference has been established for device lifetime, and no patient-reported outcomes were collected, so whether differences of this magnitude matter to patients cannot be determined here.

Just under half of our patients (47.6%) had received radiotherapy or chemoradiotherapy before laryngectomy and 57.3% had a smoking history. The relationship between irradiation and device lifetime is not consistently reported: Boscolo-Rizzo et al. found a shorter mean in situ lifetime in irradiated patients, 163.3 versus 202.9 days, with irradiation and endoscopically confirmed reflux both significant on multivariate analysis [15], whereas Lewin et al., in 3648 prostheses, found that neither radiotherapy nor the extent of surgery had a meaningful impact on device life [13]. Our data support the former. The magnitude we observed, −38.3 days, is close to the approximately 40-day difference reported in that study, and the gradient across primary surgery, radiotherapy alone, and chemoradiotherapy reproduces the observation of Jira et al. that adjuvant chemoradiotherapy rather than radiotherapy alone predisposes to a shortened device life [16]. That an ordered exposure produces an ordered response is the strongest evidence available in a design of this kind, although it remains observational and no causal claim is made.

Two features of this association bear on how it should be used. First, irradiated patients carried a higher cumulative replacement burden, so the replacement burden lies downstream of irradiation rather than confounding it; the two should not be adjusted for one another, and the marker of shorter intervals that we report in Section 3.1 is in part a marker of prior oncological treatment. Second, the effect is large enough to matter clinically: a median device survival of 84 days after chemoradiotherapy against 179 days after primary surgery corresponds to roughly twice the annual number of exchanges, with the attendant burden on patients and on outpatient capacity. Prior treatment is known before the first prosthesis is fitted, which makes it usable for planning follow-up intensity in a way that replacement burden, which can only be observed retrospectively, is not.

These findings do not establish a benefit from scheduled replacement, and the recurrent-event analysis in Section 3.7 provides no evidence of one. Elective and unscheduled procedures are not exchangeable: an elective exchange occurs by definition in a device that survived to a scheduled appointment, whereas an unscheduled exchange occurs because a device failed. A positive coefficient for elective status is therefore, in part, guaranteed by this conditioning, a form of immortal-time bias that cannot be removed by covariate adjustment. Its magnitude can, however, be estimated: modeling the status of the procedure that placed each device rather than the one that removed it returned +15.30 days, not significant and less than half the primary estimate, and the recurrent-event model returned no association at all. We therefore attribute a substantial part of the association reported in Table 2 to conditioning rather than to scheduling.

The published evidence is divided. Okła et al. reported a fall in tracheoesophageal fistula-related complications from 47% to 6%, a relative risk reduction of approximately 87%, after a Polish center moved to three-monthly replacement [21], whereas Heirman et al. concluded, from a Monte Carlo simulation at the Netherlands Cancer Institute, that fixed scheduling is not feasible, with a median of only about 25% of leakages being preventable and capturing most requiring exchange roughly every 38 days [22]. Our data do not resolve this disagreement, and one of our own findings supports the skeptical position: the between-patient variance we observed, an intraclass correlation of 0.55, is precisely the heterogeneity on which the case against fixed scheduling rests.

Transprosthetic leakage accounted for approximately half of unscheduled exchanges, periprosthetic leakage for a quarter, and prosthesis dysfunction without phonation for a fifth, proportions consistent with published series [25,26].

The predominance of transprosthetic leakage is consistent with the mechanism established in the literature, although this study contains no direct evidence of it. Silicone is rapidly colonized by a mixed bacterial and fungal biofilm in which Candida species predominate, impairing valve closure and degrading the material [18,19,20,27]; the rising Weibull hazard in Section 3.7 is compatible with such a progressive process. No cultures or microscopy were performed, and explanted devices were discarded, so the mechanism is inferred rather than demonstrated.

### Limitations

Several limitations qualify these findings. The design is retrospective and single-center, with no external validation cohort, and the modest cohort size limits its power, particularly for rare complications and for interaction and subgroup analyses. Procedural status was determined by clinical circumstance rather than allocation, and the resulting immortal-time bias cannot be removed by adjustment. The two-tier data structure introduces prevalent-user bias, as patients who died, underwent fistula closure, or abandoned a prosthetic voice before December 2024 are unrepresented, so the complication rate of 4.8% is a lower bound.

Tumor stage, diabetes, and nutritional status were not retrievable. Prior irradiation, smoking history, and proton-pump inhibitor therapy were analyzed at the patient level (Section 3.8) but did not vary within a patient, so no within-patient contrast is available for them, and their estimates rest entirely on between-patient comparison. Reflux was captured only as a prescription for proton-pump inhibitor therapy, which misclassifies in both directions and identifies treated rather than untreated disease. A limitation of a different kind applies to replacement burden itself. This variable is a patient-level summary of the entire observed event process and therefore cannot, despite the framing of the recurrent-event model, be measured at the start of an interval. Over a fixed observation window, the total number of procedures and the mean gap between them are related by construction, so the association we report is in part definitional rather than empirical. The collapse of the frailty variance once this variable is entered (θ = 0.031; Section 3.7) is consistent with it acting as a proxy for the patient-level event rate rather than as an independent predictor of it, and the within-patient analysis in Section 3.4, which is free of this problem, was directionally consistent but not significant (−4.61 days; *p* = 0.672). We therefore present replacement burden as a descriptive marker identifying patients with a high-consumption phenotype, and not as a determinant of device lifetime. No microbiological assessment of explanted devices was undertaken, and no patient-reported outcomes or inter-rater reliability data were collected.

Aspiration, pneumonia, and unplanned admissions were not systematically ascertained: all procedures were day-case and patients presenting later would typically attend primary care, so their absence reflects incomplete ascertainment as much as the absence of events. This bears directly on the central question because aspiration through an incompetent valve is the principal pathway by which leakage causes harm. Only Provox Vega and Vega XtraSeal devices were used, reflecting national reimbursement, and the device removed at each procedure could be identified only for the 65 procedures preceded by a documented replacement within the dataset; the remaining 82 were each patient’s first in the observation window. The comparison of size change between elective and unscheduled procedures is subject to the same conditioning as the interval comparison, because periprosthetic leakage is both a frequent reason for unscheduled presentation and an indication for a change in size or for an XtraSeal device; no inference about the effect of scheduling on the need for size adjustment is therefore drawn.

In summary, this analysis does not support the proposition that scheduled replacement prolongs device life. It identifies replacement burden as a robust marker of shorter intervals and outpatient exchange as safe. Whether scheduling benefits patients with a high replacement burden remains an open question and requires randomized comparison. Device design offers a further lever, with problem-solving and custom prostheses having been developed for recurrent early failure [28,29,30], as does targeted antimicrobial management [31]; voice-related quality of life is the outcome against which any strategy should ultimately be judged [32], and listener-based assessment indicates that prosthetic speech is preferred to electrolaryngeal speech in terms of sound quality, intelligibility, and prosody (Raiff et al. [33]).

## 5. Conclusions

This single-center study shows that intervals ending in a planned exchange were longer than those ending in an unscheduled one, and that patients with a higher cumulative burden of voice prosthesis replacement have shorter intervals to the next exchange. Neither observation can be interpreted as a causal statement. The first comparison is defined in part by its own outcome: in a recurrent-event analysis measuring procedural status at the start of each interval and retaining censored intervals, elective replacement showed no association with device lifetime. The second is a description rather than an effect, because cumulative burden summarizes the same event process that generates the interval and cannot be measured before it; it is reported here as a phenotype marker identifying patients who replace often. These data therefore do not support the proposition that scheduled replacement prolongs device life. What they support is the identification of patients at risk of frequent exchange. Prior oncological treatment is the clearest such marker: chemoradiotherapy before laryngectomy was associated with a median device survival of 84 days versus 179 days after primary surgery, with radiotherapy alone intermediate at 136 days. Because this information is available before the first prosthesis is fitted, it can inform the intensity of follow-up in a way that replacement burden, observable only in retrospect, cannot. Outpatient exchange was safe throughout.

Despite this low complication rate, the substantial between-patient variability in replacement intervals highlights the importance of individualized yet protocol-driven management. Future multi-center prospective studies are needed to define the optimal replacement schedule and to determine whether routine planned exchanges confer clinical benefit.

## Figures and Tables

**Table 1:** Baseline characteristics of the study population. Percentages for removed device size, change in device, and change in type were calculated based on the 65 procedures preceded by a documented replacement within the dataset, because the device removed, and hence any change relative to it, can be established only from the paired before-and-after record; a change in device denotes any difference in size or type from the device removed; all other percentages use the stated denominator of 82 patients or 147 procedures. Prior oncological treatment and smoking history are reported per patient (*n* = 82).

Variable	
Number of patients	82
Number of procedures	147
Procedures within the study window—median (IQR); mean (SD); range [patient-level, *n* = 82]	2 (1–2); 1.8 (0.8); 1–4
Cumulative replacement burden—median (IQR); mean (SD); range [patient-level, *n* = 82]	8 (5–13); 8.9 (5.3); 1–28
Age, years—median (IQR); mean (SD); range [patient-level, *n* = 82]	68 (62–73); 67.6 (8.2); 44–83
Female, *n* (%)	21 (25.6)
Prior oncological treatment, *n* (%)	
Radiotherapy alone	25 (30.5)
Chemoradiotherapy	14 (17.1)
Any prior (chemo)radiotherapy	39 (47.6)
Primary surgery, no prior (chemo)radiotherapy	43 (52.4)
Smoking history, *n* (%)	
Ever-smoker	47 (57.3)
Never-smoker	35 (42.7)
Proton-pump inhibitor therapy for reflux, *n* (%)	18 (22.0)
Removed device size, *n* (%)	
PV 4 mm	2 (3.1)
PV 6 mm	16 (24.6)
PV 8 mm	12 (18.5)
PV 10 mm	6 (9.2)
PV 12.5 mm	0 (0.0)
PVXS 6 mm	11 (16.9)
PVXS 8 mm	8 (12.3)
PVXS 10 mm	7 (10.8)
PVXS 12.5 mm	3 (4.6)
New size, *n* (%)	
PV 4 mm	4 (2.7)
PV 6 mm	42 (28.6)
PV 8 mm	32 (21.8)
PV 10 mm	10 (6.8)
PV 12.5 mm	1 (0.7)
PVXS 6 mm	16 (10.9)
PVXS 8 mm	25 (17.0)
PVXS 10 mm	13 (8.8)
PVXS 12.5 mm	4 (2.7)
Reason for replacement, *n* (%)	
Scheduled	74 (50.3)
Side leakage	18 (12.2)
Central leakage	36 (24.5)
Prosthesis dysfunction/no phonation	14 (9.5)
Other	5 (3.4)
Complications, *n* (%)	7 (4.8)
Change in device at replacement, *n* (%)	31 (47.7)
Change in type (PV vs. PVXS), *n* (%)	13 (20.0)

**Table 2:** Mixed-effects linear regression analysis of factors associated with time between prosthesis replacements. Random-intercept model fitted to 65 procedures in 50 patients. All four a priori covariates were adjusted simultaneously. Coefficients express the change in the replacement interval in days. Sensitivity specifications are reported separately in Table 3.

Variable	β Coefficient (95% CI)	*p*-Value
Cumulative replacement burden, per additional lifetime exchange	−3.70 (−5.84 to −1.57)	0.001
Scheduled replacement	35.47 (15.80 to 55.14)	<0.001
Covariate: age, per year	0.50 (−0.91 to 1.91)	0.489
Covariate: female sex	−10.47 (−37.01 to 16.07)	0.439

**Table 3:** Stability of the two primary associations across sensitivity specifications. All models are random-intercept mixed-effects models adjusted for age and sex. Coefficients express the change in replacement interval in days. Rows indented beneath the primary model are covariates of that model, not separate specifications; *n* is given for every specification.

Specification	Cumulative Replacement Burden, β (95% CI)	Elective Status, β (95% CI)
Primary model (65 procedures)	−3.70 (−5.84 to −1.57)	35.47 (15.80 to 55.14)
—Age, per year	0.50 (−0.91 to 1.91); *p* = 0.489	—
—Female sex	−10.47 (−37.01 to 16.07); *p* = 0.439	—
Excluding frequent replacers, interval criterion (41 procedures)	−1.97 (−4.67 to 0.72)	13.38 (−5.26 to 32.02)
Winsorized at 5th/95th percentile (65 procedures)	−3.39 (−5.43 to −1.34)	34.45 (15.34 to 53.55)
Adjusted for duration of rehabilitation (65 procedures)	−5.10 (−8.86 to −1.33)	34.51 (13.54 to 55.47)
Adjusted for operator volume (65 procedures)	−3.95 (−6.20 to −1.70)	33.39 (12.23 to 54.55)
Status of the placing procedure (65 procedures)	−4.46 (−6.76 to −2.16)	15.30 (−6.85 to 37.45)
Leave-one-patient-out (50 models, 62–64 procedures each)	−4.55 to −3.32	26.06 to 40.52

## Data Availability

The source clinical records of laryngectomized patients from which this study was derived constitute special-category personal data under Article 9 of Regulation (EU) 2016/679 (GDPR) and cannot be deposited publicly. All direct identifiers were removed before analysis, and the anonymized analytical dataset underlying the reported results is available from the corresponding author on reasonable request, subject to approval by the Medical University of Silesia and a data-sharing agreement. No individual-level data are included in this article or its Supplementary Materials.

## Supplementary Materials

The following supporting information can be downloaded at https://www.mdpi.com/article/10.3390/cancers18162545/s1. Table S1: STROBE statement—checklist of items that should be included in reports of cohort studies.

## Abbreviations

The following abbreviations are used in this manuscript:

PV	Provox Vega
PVXS	Provox Vega XtraSeal
TEP	Tracheoesophageal puncture
ICC	Intraclass correlation coefficient
OR	Odds ratio
CI	Confidence interval
SD	Standard deviation
IQR	Interquartile range
RT	Radiotherapy
CRT	Chemoradiotherapy
GERD	Gastro-esophageal reflux disease
PROM	Patient-reported outcome measure
