# Supplementary material for: Factors Associated with Replacement Interval and Complications of Provox Voice Prostheses in Post-Laryngectomy Patients: A Single-Center Retrospective Recurrent-Event Analysis
Source: Cancers (Basel). 2026 Aug 7;18(16):2545. doi: 10.3390/cancers18162545 (PMC13511052; doi:10.3390/cancers18162545)
Supplement: Supplementary file 1 [file cancers-18-02545-s001.zip › cancers-4473385-Supplementary Table S1.pdf]

STROBE Statement—

Checklist of items that should be included in reports of cohort studies

Manuscript ID: cancers-4473385

Factors Associated with Replacement Interval and Complications of Provox Voice Prostheses in Post-Laryngectomy Patients: A Single-Center Retrospective Recurrent-Event Analysis

Page and line numbers refer to the revised manuscript cancers-4473385_REV7.docx submitted with this checklist, as rendered to PDF; line numbering is continuous and excludes tables, headers and footers.

Item	No.	Recommendation	Page / line
**Title and abstract**			
Title and abstract	1a	Indicate the study design with a commonly used term in the title or the abstract	Title, p. 1, lines 3–5; Abstract, p. 1, lines 28–31
	1b	Provide in the abstract an informative and balanced summary of what was done and what was found	Simple Summary, p. 1, lines 14–27; Abstract, p. 1–2, lines 28–51
**Introduction**			
Background / rationale	2	Explain the scientific background and rationale for the investigation being reported	p. 2–4, lines 56–121
Objectives	3	State specific objectives, including any prespecified hypotheses	p. 4, lines 122–129
**Methods**			
Study design	4	Present key elements of study design early in the	Section 2, p. 4–5, lines 130–146; Section 2.1, p. 5, lines 147–162; Figure 1, p. 6, lines 164–165

Item	No.	Recommendation	Page / line
		paper	
Setting	5	Describe the setting, locations, and relevant dates, including periods of recruitment, exposure, follow-up, and data collection	Section 2.1, p. 5, lines 147–162 (analytical dataset June 2024 – February 2026, 145 of 147 procedures from December 2024; institutional log May 2020 – March 2026; census date 20 February 2026)
Participants	6a	Give the eligibility criteria, and the sources and methods of selection of participants. Describe methods of follow-up	Section 2.1, p. 5–7, lines 147–191; Figure 1, p. 6, lines 164–165
	6b	For matched studies, give matching criteria and number of exposed and unexposed	Not applicable
Variables	7	Clearly define all outcomes, exposures, predictors, potential confounders, and effect modifiers. Give diagnostic criteria, if applicable	Section 2.1, p. 6–7, lines 166–177 (the two counts of replacement activity are defined and distinguished here); Section 2.2, p. 9–10, lines 200–220; Section 2.3, p. 10–11, lines 221–263. Prior-treatment categories defined in Section 3.8, p. 22, lines 492–497
Data sources / measurement	8	For each variable of interest, give sources of data and details of methods of assessment. Describe comparability of assessment methods if there is more than one group	Section 2, p. 4–5, lines 130–146; Section 2.1, p. 5–7, lines 147–191; Section 2.2, p. 9–10, lines 200–220. Prior oncological treatment, smoking history and proton-pump inhibitor therapy were abstracted from the departmental clinical record; reflux was captured only as a prescription for proton-pump inhibitor therapy, a proxy discussed in Section 3.8, p. 24, lines 545–556
Bias	9	Describe any efforts to address potential sources of bias	Section 2.1, p. 7, lines 178–191; Section 2.3, p. 10–11, lines 228–263; Section 3.6, p. 17–18, lines 409–429 (left truncation); Section 3.7, p. 18–22, lines 430–490 (conditioning, and the exclusion of cumulative burden from the primary specification); Section 3.8, p. 22–25, lines 492–564, including the differential contribution of irradiated and non-irradiated patients at lines

Item	No.	Recommendation	Page / line
			502–510; Limitations, p. 27–28, lines 641–683
Study size	10	Explain how the study size was arrived at	Section 2.1, p. 5, lines 147–162 (complete consecutive census of the structured-documentation period). Precision of the patient-level exposure estimates is conveyed by the confidence intervals reported in Section 3.8, p. 24–25, lines 560–564
Quantitative variables	11	Explain how quantitative variables were handled in the analyses. If applicable, describe which groupings were chosen and why	Section 2.1, p. 6–7, lines 166–177; Section 2.3, p. 10–11, lines 221–263; Section 3.5, p. 16–17, lines 394–406; Section 3.8, p. 22–23, lines 492–508 (irradiation analysed both as a binary exposure and as three ordered categories)
Statistical methods	12a	Describe all statistical methods, including those used to control for confounding	Section 2.3, p. 10–11, lines 221–263. Section 3.7, p. 19–20, lines 448–471, states why cumulative replacement burden is excluded from the primary recurrent-event specification; Section 3.8, p. 23–24, lines 520–544, states why it is treated as a mediator of prior irradiation and not adjusted for
	12b	Describe any methods used to examine subgroups and interactions	Section 2.3, p. 10–11, lines 237–263; Section 3.3, p. 14, lines 343–356; Section 3.5, p. 16–17, lines 394–406; Section 3.7, p. 19–20, lines 448–471; Section 3.8, p. 22–24, lines 492–556
	12c	Explain how missing data were addressed	Section 3.1, p. 12, lines 279–285 — the reduction from 147 to 65 procedures is structural (82 procedures were each patient's first and have no preceding interval); no covariate value was missing; 32 patients contributed a single procedure. Patient-level exposures in Section 3.8 were complete for all 82 patients, although only 50 contribute a completed interval and that subset is unbalanced with respect to irradiation (Section 3.8, p. 23, lines 509–517)
	12d	If applicable, explain how loss to follow-up was addressed	Section 2.3, p. 11, lines 245–253; Section 3.7, p. 18–19, lines 430–445 (81 right-censored intervals retained; one interval of zero length excluded, giving 146)
	12e	Describe any sensitivity	Section 3.4, p. 14–16, lines 357–393; Section 3.6,

Item	No.	Recommendation	Page / line
		analyses	p. 17–18, lines 409–429; Section 3.7, p. 18–22, lines 430–490; Section 3.8, p. 22–25, lines 492–564; Table 3, p. 15, lines 386–387
**Results**			
Participants	13a	Report numbers of individuals at each stage of study	Section 3.1, p. 11–12, lines 262–285; Figure 1, p. 6, lines 164–165
	13b	Give reasons for non-participation at each stage	Figure 1, p. 6, lines 164–165; Section 2.1, p. 7, lines 180–185
	13c	Consider use of a flow diagram	Figure 1, p. 6, lines 164–165
Descriptive data	14a	Give characteristics of study participants and information on exposures and potential confounders	Table 1, p. 7–9 (caption p. 7, lines 192–198); Section 2.1, p. 7, lines 186–191; Section 3.8, p. 22, lines 492–497. Table 1 reports both counts of replacement activity: procedures within the study window and cumulative replacement burden
	14b	Indicate number of participants with missing data for each variable of interest	Table 1 caption, p. 7, lines 192–198; Section 3.1, p. 12, lines 279–285; Section 3.8, p. 23, lines 509–517; Limitations, p. 28, lines 674–678 (the device removed at a procedure is recorded only for the 65 procedures preceded by a documented replacement within the dataset; no covariate value was missing)
	14c	Summarise follow-up time	Section 3.6, p. 17–18, lines 409–417; Section 3.7, p. 18, lines 430–437
Outcome data	15	Report numbers of outcome events or summary measures over time	Section 3.1, p. 11–12, lines 262–301; Section 3.2, p. 13, lines 302–334; Section 3.7, p. 18–22, lines 430–490; Section 3.8, p. 22–25, lines 492–564; Table 1, p. 7–9 (caption p. 7, lines 192–198); Table 2, p. 12, lines 298–301; Figures 3 and 6, p. 18 and p. 23
Main results	16a	Give unadjusted estimates and, if applicable, confounder-adjusted estimates and	Table 2, p. 12, lines 298–301 (primary linear model with all covariates); Table 3, p. 15, lines 386–387 (every sensitivity specification, with n stated for each); Section 3.7, p. 19–20, lines 448–471 (primary

Item	No.	Recommendation	Page / line
		their precision. Make clear which confounders were adjusted for and why they were included	recurrent-event model without cumulative burden, and the secondary descriptive model with it); Section 3.8, p. 22–23, lines 498–517, which reports unadjusted group means alongside estimates adjusted for age, sex, proton-pump inhibitor therapy and smoking history, and states the selection to which both are subject
	16b	Report category boundaries when continuous variables were categorized	Section 2.3, p. 10–11, lines 237–263; Section 3.5, p. 16, lines 394–400 (age dichotomised at 65 years); Section 3.8, p. 22, lines 492–497 (three mutually exclusive prior-treatment categories)
	16c	If relevant, consider translating estimates of relative risk into absolute risk for a meaningful time period	Section 3.7, p. 18–19, lines 436–445 and Figure 3, p. 19, lines 446–447 (Kaplan–Meier survival: 78.5% at 90 days, 64.7% at 120 days, 26.4% at 180 days, 9.2% at 270 days, with 95% confidence intervals); Section 3.8, p. 23, lines 518–520 and Figure 6, p. 25, lines 565–574 (median device survival 179, 136 and 84 days by prior-treatment category; the caption states the unit of n and the independence assumption)
Other analyses	17	Report other analyses done — eg analyses of subgroups and interactions, and sensitivity analyses	Sections 3.3–3.8, p. 14–25, lines 343–564; Figures 2, 4, 5 and 6, p. 17, 20, 21 and 23; Tables 2 and 3, p. 12 and p. 15
**Discussion**			
Key results	18	Summarise key results with reference to study objectives	Section 4, p. 25–26, lines 575–602
Limitations	19	Discuss limitations of the study, taking into account sources of potential bias or imprecision. Discuss both direction and magnitude of any potential bias	Limitations, p. 27–28, lines 641–691. Magnitude of the conditioning artefact affecting procedural status is estimated in Section 3.4, p. 15, lines 381–387 and Section 3.7, p. 19–20, lines 448–479; the analogous limitation affecting cumulative replacement burden, and the reason the frailty collapse is not interpreted causally, are stated at p. 28, lines 655–667; the misclassification inherent in using proton-pump inhibitor prescription as a proxy for reflux is stated in Section 3.8, p. 24–25,

Item	No.	Recommendation	Page / line
			lines 545–564 and in Limitations, p. 27–28, lines 653–655
Interpretation	20	Give a cautious overall interpretation of results considering objectives, limitations, multiplicity of analyses, results from similar studies, and other relevant evidence	Section 4, p. 25–28, lines 575–691; Section 5, p. 29, lines 692–720. Concordance of the irradiation finding with Boscolo-Rizzo et al. [15] and Jira et al. [16] is discussed at p. 26, line 591
Generalisability	21	Discuss the generalisability (external validity) of the study results	Limitations, p. 27, line 641
**Other information**			
Funding	22	Give the source of funding and the role of the funders for the present study and, if applicable, for the original study on which the present article is based	Funding statement, p. 30, line 727: this research received no external funding

Note. An Explanation and Elaboration article discusses each checklist item and gives methodological background and published examples of transparent reporting. The STROBE checklist is best used in conjunction with this article, which is freely available on the websites of *PLoS Medicine*, *Annals of Internal Medicine* and *Epidemiology*. Information on the STROBE Initiative is available at www.strobe-statement.org.
